# Impact of deploying multiple point-of-care tests with a ‘sample first’ approach on a sexual health clinical care pathway. A service evaluation

**DOI:** 10.1136/sextrans-2016-052988

**Published:** 2017-02-03

**Authors:** Emma M Harding-Esch, Achyuta V Nori, Aseel Hegazi, Marcus J Pond, Olanike Okolo, Anthony Nardone, Catherine M Lowndes, Phillip Hay, S Tariq Sadiq

**Affiliations:** 1 Applied Diagnostic Research and Evaluation Unit, St George's University of London, Institute for Infection & Immunity, London, UK; 2 HIV/STI Department, National Infection Service, Public Health England, London, UK; 3 Courtyard Clinic, St George's University Hospitals NHS Foundation Trust, London, UK

**Keywords:** CLINICAL STI CARE, SERVICE DELIVERY, DIAGNOSIS, BACTERIAL INFECTION, TRICHOMONAS

## Abstract

**Objectives:**

To assess clinical service value of STI point-of-care test (POCT) use in a ‘sample first’ clinical pathway (patients providing samples on arrival at clinic, before clinician consultation). Specific outcomes were: patient acceptability; whether a rapid nucleic acid amplification test (NAAT) for *Chlamydia trachomatis/Neisseria gonorrhoeae* (CT/NG) could be used as a POCT in practice; feasibility of non-NAAT POCT implementation for *Trichomonas vaginalis* (TV) and bacterial vaginosis (BV); impact on patient diagnosis and treatment.

**Methods:**

Service evaluation in a south London sexual health clinic. Symptomatic female and male patients and sexual contacts of CT/NG-positive individuals provided samples for diagnostic testing on clinic arrival, prior to clinical consultation. Tests included routine culture and microscopy; CT/NG (GeneXpert) NAAT; non-NAAT POCTs for TV and BV.

**Results:**

All 70 (35 males, 35 females) patients approached participated. The ‘sample first’ pathway was acceptable, with >90% reporting they were happy to give samples on arrival and receive results in the same visit. Non-NAAT POCT results were available for all patients prior to leaving clinic; rapid CT/NG results were available for only 21.4% (15/70; 5 males, 10 females) of patients prior to leaving clinic. Known negative CT/NG results led to two females avoiding presumptive treatment, and one male receiving treatment directed at possible *Mycoplasma genitalium* infection causing non-gonococcal urethritis. Non-NAAT POCTs detected more positives than routine microscopy (TV 3 vs 2; BV 24 vs 7), resulting in more patients receiving treatment.

**Conclusions:**

A ‘sample first’ clinical pathway to enable multiple POCT use was acceptable to patients and feasible in a busy sexual health clinic, but rapid CT/NG processing time was too long to enable POCT use. There is need for further development to improve test processing times to enable POC use of rapid NAATs.

## Introduction

STIs, which continue to present a large public health burden in England, with more than 400 000 STI diagnoses a year,^1^ are largely asymptomatic,^2^ resulting in long infectious periods. Regular STI testing and treatment aim to reduce these infectious periods and onward transmission rates but there is presently no evidence of overall reductions in diagnosis rates from national STI testing programmes such as the National Chlamydia Screening Programme (NCSP).^1^ ^3^

Sexual Health Clinics (SHCs) in England report median times between testing and treatment for *Chlamydia trachomatis* (CT) of 8 days,^4^ while the NCSP reports 94% of CT infections being treated within 30 days.^5^ Sex with new partners has been noted in the period between testing and receiving treatment for CT^6^ suggesting that, unless followed by prompt treatment, widespread testing may have limited impact reducing the burden of infection. Additionally, longer durations of infection may increase risks of clinical complications.^7^ Moreover, recalling patients for treatment requires significant time and financial investment for SHCs.^4^

Implementing rapid tests, where results are available within 2 hours of sample provision,^8^ as point-of-care tests (POCTs), where tests are conducted and results and treatment given within one consultation,^9^ offers possibilities of deploying STI test-and-treat strategies, enabling both rapid and personalised STI treatments while improving antibiotic stewardship. Innovations in rapid nucleic acid amplification tests (NAATs) and non-NAATs may provide improvements and alternatives to microscopy, the main STI point-of-care test (POCT) available in SHCs.^10^ However, there are few studies evaluating use of rapid and POC tests in practice, including how patients and providers respond to them, and time taken between test, result and treatment.^11^ ^12^

We performed a service evaluation of a modified, so-called ‘sample first’ (patients providing samples on arrival at clinic, before clinician consultation) clinical care pathway to assess feasibility and acceptability of implementing POCTs for symptomatic patients in an SHC, and to evaluate impact of the pathway on patient management.

Specific objectives were to assess:

1. Acceptability to patients of a ‘sample first’ clinical pathway to enable POCT use.
2. Whether a ‘sample first’ approach enabled a rapid NAAT to be used as a POCT in practice.
3. Whether a ‘sample first’ approach was feasible for non-NAAT POCT use in practice.
4. The impact on patient diagnosis and treatment of multiple POCTs within a ‘sample first’ approach.

## Methods

The service evaluation was performed between January and April 2013 at the St George's University Hospitals NHS Foundation Trust Sexual Health Clinic. Tests were donated by manufacturers, limiting sample size to 70 patients (35 males and 35 females). Following review by the Interim Research Governance Lead of the Joint Research Office, St George's, the project was determined as fulfilling the criteria of a ‘service evaluation’. Ethics submission and approval was therefore not a requirement. This manuscript was written following Standards for Quality Improvement Reporting Excellence (SQUIRE) guidelines (see online supplementary material 1).^13^

10.1136/sextrans-2016-052988.supp1 supplementary material

Tests, all of which were CE-marked, included: *CT/Neisseria gonorrhoeae* (CT/NG) NAAT (Cepheid GeneXpert, California, USA) and lateral flow tests for *Trichomonas vaginalis* (TV) (OSOM Trichomonas Rapid Test, Sekisui Diagnostics, Massachusetts, USA; an antigen-detection immunochromatographic enzyme immunoassay) and bacterial vaginosis (BV) (Alere VS-SENSE, Massachusetts, USA). The GeneXpert CT/NG NAAT achieves sensitivities and specificities >97% for urogenital samples compared with other NAATs,^14^ ^15^ and is currently widely used as a rapid test, but not a POCT.^16^ OSOM TV has reported sensitivities of 88.2%–98.0% and specificities of 99.4%–100% compared with wet mount microscopy, culture or a composite gold standard including PCR assays.^17–19^ The Alere VS-SENSE BV test, an acidity test based on pH levels designed to be used together with additional clinical data/indications such as Amsel or Hay-Ison criteria or Nugent Gram stain,^20^ has reported sensitivities of 82.3%–91% and specificities of 94.2%–97.8% compared with pH paper.^21^ ^22^ On the basis of the tests' intention for use, clinicians were informed to use the GeneXpert CT/NG and OSOM TV tests as diagnostic tests with definitive results. For BV, diagnosis was based on Hay-Ison criteria of vaginal flora: grade III was designated BV, grades 0 and I were designated not BV and grade II was designated mixed flora where a clinical decision to treat as BV could be made (see online supplementary material 2). Clinicians were informed that the VS-SENSE BV test was a pH-based assay, and as such could be used as a diagnostic aid to complement normal clinical diagnosis.

10.1136/sextrans-2016-052988.supp2 supplementary material

Criteria for inclusion in the pathway, deliberately selected to increase likelihood of having CT/NG-positive patients to enable worthwhile impact assessment on patient management, were: patients aged ≥16 years; males not having passed urine in preceding 2 hours; symptomatic (males with symptoms suggestive of urethritis; females with vaginal discharge, pelvic pain, dyspareunia, intermenstrual bleeding or postcoital bleeding) or sexual contacts of CT/NG-positive individuals. Exclusion criteria were patients not fulfilling the inclusion criteria, and patients with complex symptoms or histories.

**Figure 1:**
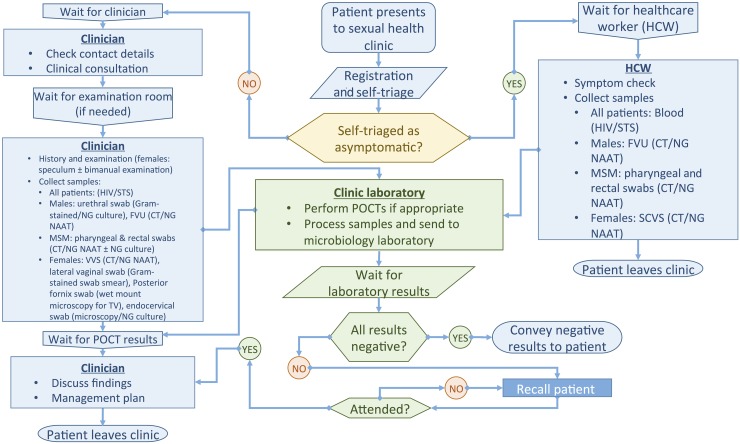
St George's University Hospitals NHS Foundation Trust Sexual Health Clinic clinical pathways. Symptomatic: males (symptoms of urethritis or epididymo-orchitis): dysuria; urethral discharge; urinary discomfort, frequency, haematuria, haematospermia; scrotal lump/discomfort. Females (symptoms of vaginal discharge or pelvic inflammation): vaginal discharge, vulval/vaginal discomfort; dyspareunia/pelvic pain; postcoital and/or intermenstrual bleeding; dysuria, urinary discomfort, frequency, urgency. Contacts of gonorrhoea-positive individuals are managed as symptomatic. Contacts of chlamydia-positive individuals are managed as asymptomatic, but see a clinician as they need treatment. MSM, men who have sex with men; CT, *Chlamydia trachomatis*; NG, *Neisseria gonorrhoeae*; TV, *Trichomonas vaginalis*; FVU, first void urine; SCVS, self-collected vaginal swab; VVS, vulvovaginal swab; STS, serological tests for syphilis; NAAT, nucleic acid amplification test; POCT, point-of-care test.

The routine clinical pathway at the St George's walk-in SHC is depicted in figure 1. Briefly, patients presenting to the clinic are self-triaged at registration as asymptomatic (for an STI check-up with no other complaints or needs) or as not asymptomatic (have symptoms or other needs). Asymptomatic patients follow a ‘quick-check’ pathway, seeing non-medical staff who confirm pathway eligibility and collect appropriate samples. Symptomatic patients wait to have a consultation with a clinician, resulting in a management plan including examination and diagnostic tests. Microscopy is conducted to provide POCT results for non-gonococcal urethritis (NGU) (males) and BV and TV (females) and then treatment given based on overall clinical diagnosis. Patients are then clinically discharged. NAAT laboratory results are available in 1–14 days.

**Figure 2:**
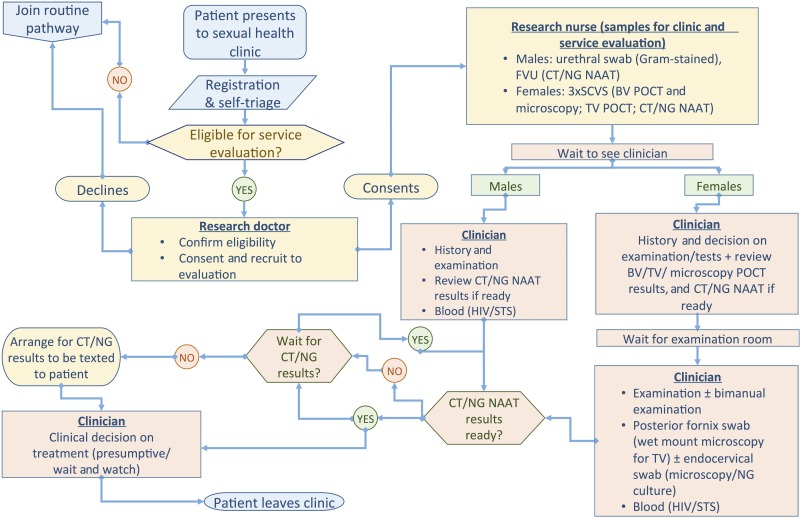
‘Sample first’ service evaluation patient pathways. CT, *Chlamydia trachomatis*; NG, *Neisseria gonorrhoeae*; TV, *Trichomonas vaginalis*; BV, bacterial vaginosis; FVU, first void urine; SCVS, self-collected vaginal swab; VVS, vulvovaginal swab; STS, serological tests for syphilis; NAAT, nucleic acid amplification test, POCT: point-of-care test.

The service evaluation modified ‘sample first’ pathway is shown in figure 2. Patients were recruited during 24 clinic sessions by a dedicated senior clinician, who reviewed patients’ routine self-triage forms, approached eligible patients and explained the modified ‘sample first’ clinical pathway. Those accepting the pathway provided samples at recruitment to increase likelihood of receiving all rapid test results during their clinic visit; had routine CT/NG NAAT laboratory testing replaced by Cepheid CT/NG GeneXpert NAAT and also had non-NAAT POCTs (OSOM TV and VS-SENSE BV) performed in addition to routine culture and microscopy.

Samples were collected in the following order. For males, a urethral swab was first collected by nursing staff, followed by a self-collected first void urine (FVU) sample. Females were asked to provide three self-collected vaginal swabs (SCVS), one each for the CT/NG, TV and BV tests, and could choose the order in which swabs were taken.

The following tests were performed in the clinic laboratory: CT/NG on FVU (males) and SCVS (females) following manufacturer's instructions (test-time, 90 min); microscopy examination of wet-mount of vaginal swab (females), and Gram-stained urethral smear (males) and vaginal smear (females) (result available within 15 min) and TV and BV POCTs (test-time 15 and 5 min, respectively). All CT/NG and positive BV/TV POCT results were verified by a senior clinician. It was not feasible to confirm BV/TV POCT negatives as results had to be read within a specific time-point.

Following sample collection, patients joined the routine clinic queue and were seen by clinicians who were made aware of any POCT results available at the time of consultation. In all other respects, patients received usual clinic management, including clinical diagnosis and treatment, health promotion and partner notification. Clinicians did not delay the patient pathway to allow for the rapid test results to become available, and only acted on results that were available at the time of final consultation, except for patients who stated at recruitment that they wanted to wait for their CT/NG results beyond their clinical consultation. These patients were then seen again and appropriately managed according to their CT/NG rapid test results.

CT/NG, TV and BV POCT and urethral smear results were manually entered into patients’ records. During the evaluation, clinic staff recorded exact timings in minutes of each stage of the ‘sample first’ pathway for each patient and waiting periods between these, that is, time of patient arrival, triage, sample collection, clinical consultation, CT/NG test result availability, discharge from clinic. Patient records were reviewed to determine diagnosis, clinical care pathway duration and treatment, and to infer how these may have differed if POCT results had not been available.

Patients were given an anonymous unlinked feedback questionnaire at the time of agreeing to undertake the ‘sample first’ pathway, to be completed before leaving clinic, which covered their opinions on length of stay in clinic, providing samples on arrival at clinic, receiving test results in the same clinical visit, quality of service received compared with any previous visits and whether they intended waiting for rapid CT/NG results in case they were not available at the time of clinical diagnosis. There was also a free-text comments box.

### Statistical analyses

Specific outcome measures were:

1. Proportion of patients who responded in favour of the ‘sample first’ approach.
2. Proportion of patients who received test results in the same clinical visit.
3. Individual durations of patient pathway component parts (triage, recruitment, sample collection, clinician consultation, discharge from clinic).
4. Comparisons of patient management using the modified ‘sample first’ pathway with that assumed if the routine pathway were followed, with regard to changes in diagnoses of TV and BV by comparing standard wet mount microscopy with the OSOM test, and Gram stain microscopy with the VS-SENSE test, respectively; NGU if CT/NG POCT results available.

## Results

Seventy (35 male, 35 female) symptomatic patients were tested using the ‘sample first’ clinical pathway (table 1). All patients approached agreed to undertake the pathway, with no refusals, and all stayed until they were discharged from clinic, whether or not rapid CT/NG results were available.

**Table 1:** Summary of test results and patient clinical pathway timings

	Males	Females	Total
Number of patients recruited	35	35	70
*Test results*			
CT/NG			
Cepheid CT positive: N (% of total)	6 (17.1)	0 (0)	6 (8.6)
Cepheid NG positive: N (% of total)	1 (2.9)	0 (0)	1 (1.4)
Non-gonococcal urethritis			
Based on Gram stain microscopy (urethral smear): N (% of male total with results)	13 (37.1)	N/A	13 (37.1)
TV			
Based on wet-mount microscopy: N (% of female total)	N/A	2 (5.7)*	2 (5.7)*
Based on OSOM: N (% of female total)	N/A	3 (8.6)*	3 (8.6)*
BV			
Based on Gram stain microscopy: N (% of female total)	N/A	7 (20.0)†,‡	7 (20.0)†,‡
Based on VS-SENSE: N (% of female total)	N/A	24 (68.6)†,§	24 (68.6)†,§
Median (range) time, in minutes, spent in clinic from:			
Clinic arrival to discharge from clinic	113 (59–206)	110 (59–184)	113 (59–206)
Clinical arrival to first clinical consultation	93 (45–182)	79 (32–144)	90 (32–182)
Clinic arrival to CT/NG test result being available¶	159 (128–216)	153 (112–249)	159 (112–249)
Sample collection to discharge from clinic	71 (30–149)	75 (42–157)	74 (30–157)
Sample collection to clinical consultation	55 (7–131)	46 (16–88)	48 (7–131)
Sample collection to CT/NG test result being available¶	107 (94–189)	106 (97–204)	107 (94–204)
Discharge from clinic to CT/NG test result being available, for patients who did not receive CT/NG while in clinic¶	39 (6–108)	127 (120–155)	46 (6–155)
CT/NG test result received			
During clinical consultation	4	8**	12
After clinical consultation, but patient waited for results before leaving clinic	1	2	3
After patient left clinic	30	25	55

*The two microscopy positives were OSOM TV positive.

†The seven microscopy positives were VS-SENSE BV positive.

‡An additional 11 were borderline BV by microscopy, of whom 6 had clinical evidence of BV.

§Two VS-SENSE BV negatives had borderline microscopy and clinical evidence of BV.

¶The time at which the CT/NG result was available was recorded for 31 patients only.

**Two females received their results but it is unknown whether they waited or not—we have assumed results were received during clinical consultation.

BV, bacterial vaginosis; CT, *Chlamydia trachomatis*; TV, *Trichomonas vaginalis*.

Microscopy and non-NAAT POCT results were all available before the end of patients’ consultations. However, only 15/70 (21.4%) (5 males, 10 females) were recorded as having received their CT/NG results before leaving clinic. For patients who were discharged before receiving their CT/NG result, the median delay between patient discharge and CT/NG result availability was 46 min (range 6–155).

The TV and BV non-NAAT POCTs detected more positives than routine clinical tests did (table 1). For TV, this resulted in one additional TV diagnosis and treatment. For BV, 11 of the 24 POCT positives (45.8%) were confirmed as BV positive (7 with microscopy grade III, 4 with mixed flora designated as BV following clinical indicators). A further three patients were TV positive and received treatment (metronidazole) that would equally have treated a BV infection. Of the remaining 10 BV POCT positives that clinically did not have BV (6 had no signs of BV and 4 had mixed flora where the symptoms and signs indicated another aetiology), 6 were treated as BV. Two females were not given presumptive treatment due to known negative CT/NG results. One male patient diagnosed with NGU by urethral smear and negative for CT/NG received treatment targeted at possible *Mycoplasma genitalium* (MG) infection. No other treatment changes were made based on POCT results in men.

Of the 70 patients following the ‘sample first’ pathway, 24 completed the anonymous questionnaire after clinic discharge (table 2). Over 90% of those answering the survey were happy to give samples on arrival, and liked the idea of having test results in the same clinical visit. A number of patients expressed satisfaction in free-text comments of the more efficient service that reduced their anxiety. All patients who were in clinic ≤2 hours found the amount of time in clinic acceptable.

**Table 2:** Anonymous feedback questionnaire responses, by duration of patient clinic visit

	Time in clinic (n/N, %)			Total (N=24)
	<1 hour (N=10)	1–2 hours (N=11)	>2 hours (N=3)	
Amount of time in clinic was acceptable	9/9 (100.0)	10/10 (100.0)	1/3 (33.3)	20/22 (90.9)
‘Sample first’ approach was acceptable	9/10 (90.0)	11/11 (100.0)	3/3 (100.0)	23/24 (95.8)
Liked idea of having results in same clinical visit	8/10 (80.0)	11/11 (100.0)	3/3 (100.0)	22/24 (91.7)
Were waiting for CT/NG test results beyond clinical consultation	9/10 (90.0)	4/10 (40.0)	2/3 (66.7)	15/23 (65.2)
Found service better than at previous visits	5/9 (56.0)*	8/9 (88.9)	1/1 (100.0)	14/19 (73.7)

*The remaining 4/9 (44.4%) found the service to be the same as before.

CT/NG, *Chlamydia trachomatis/Neisseria gonorrhoeae*.

## Discussion

This ‘sample first’ pathway, which enabled the real-life impact of rapid and POC test introduction into our SHC, was highly acceptable, demonstrated by >90% of those providing feedback in favour of giving samples on arrival and receiving results in the same clinical visit.

This is the first report of absolute times *waited* for the CT/NG GeneXpert, as opposed to *willingness* to wait.^16^ ^23^ Results from microscopy and non-NAAT POCTs were all available by time of consultation and compared with the standard clinical pathway, resulted in increased numbers of diagnoses at first visit, compared with routine microscopy alone, leading to an increased number of treatments given. This had potential benefits of reducing duration of infection and risk of transmission to sexual partners.

In stark contrast, CT/NG results were available in time for only a fifth of patients, with GeneXpert processing times too long to impact on the majority of patients’ clinical care, a finding consistent with previous reports.^12^ ^16^ Nevertheless, the ‘sample first’ approach enabled changed patient management for some of the 15 patients who did receive their CT/NG results in time (two females avoided presumptive treatment and one male received treatment targeted at presumed MG, following negative CT/NG results), demonstrating the potential role of POCTs in antibiotic stewardship.^24^

The main limitations of this evaluation are the small sample size, and only 44.3% of patients having had accurate timings of the pathway recorded due to a paper-based clinical notes system in use at the time. In addition, as this was a service evaluation rather than a research study we did not set out to assess diagnostic accuracy.

The ‘sample first’ pathway was a simplification of the routine clinic pathway, which led to the relative successful deployment of POCTs. Alternative pathways that might ensure patients receive CT/NG results during, or soon after, clinical consultation^25^ are unlikely to be effective in our clinic given that the median time between patient arrival and clinical consultation is equivalent to the GeneXpert 90 min test-time, even if patients self-collected samples prior to clinic arrival. For our clinic, an ideal CT/NG NAAT POCT turnaround time using a ‘sample first’ pathway would need to be no more than approximately 30 min, given the median time between sample collection and clinical consultation of 48 min. There are some promising technologies in development, but not yet readily available, that may fulfil this requirement while maintaining high diagnostic accuracy.^11^

Although we used CE-marked tests, regulatory approval does not guarantee high accuracy,^26^ and the degree to which accuracy can be compromised in the interests of reducing turnaround time is context-dependent.^27^ ^28^ The GeneXpert, already shown to be highly accurate,^14^ ^15^ is now used routinely in a number of non-POCT settings.^16^ The extra TV diagnosis is consistent with other reports of high OSOM TV test sensitivity,^17^ ^18^ ^29^ whereas the six BV cases that were diagnosed by the POCT alone, and consequently treated, are more difficult to interpret as the few performance evaluations of VS-SENSE use pH paper as the reference standard,^21^ ^22^ and results should be interpreted together with clinical examination.^20^ In our service evaluation, it would appear that in some instances, the VS-SENSE BV POCT was used as a diagnostic tool as opposed to as a diagnostic aid, as had been instructed. This may have been due to the manner in which the BV POCT result was reported on clinical notes, as either BV POCT ‘positive’ or ‘negative’, which implies a definitive result. Not having a full understanding of the clinicians’ decision-making process with regard to the BV POCT result is a limitation of this study. To help our understanding of how to introduce new diagnostic tests in SHCs, qualitative research investigating healthcare professionals’ views towards novel diagnostic technologies is warranted.

Our findings show that multiple POCTs deployed in a ‘sample first’ SHC clinical pathway have potential to affect patient treatment and management, with additional TV and BV diagnoses and three patients not receiving presumptive treatment. However, patients were unwilling to wait >2 hours for CT/NG GeneXpert NAAT results, and with only 21.4% of patients receiving their CT/NG results before leaving clinic, this test cannot be regarded as a POCT for our clinical patient population. New diagnostic tests with faster turnaround times, ideally of around 30 min, without compromising on diagnostic accuracy, are needed to enable test-and-treat strategies to be implemented in SHCs, as well as in other STI testing settings.

Test-and-treat strategies have potential advantages such as: immediate and accurate management of patients resulting in less onward transmission and reduced risk of development of sequelae; reduced use of presumptive or inappropriate treatment thus decreasing the risk of antimicrobial resistance development; less patient loss to follow-up and improved partner notification.^25^ However, the adoption by SHCs of POCTs that enable test-and-treat strategies is also dependent on the cost of the tests, and the funding structures in place. Sexual health commissioning in England is complex, with regional, provider and reimbursement type (tariff vs block) differences.^30^ Furthermore, introduction of POCTs in SHCs for organisms, such as CT and NG, which are currently being tested for on high-throughput NAAT platforms in centralised laboratories, would require SHCs to be convinced of the value for money of POCT introduction. In these times of budget constraints, the ‘effectiveness’ of POCTs must outweigh the additional costs to SHCs. Although an initial cost-effectiveness analysis of the CT/NG GeneXpert in the UK SHCs provided promising results,^25^ the specific ‘sample first’ pathway we employed was not modelled. Further cost-effectiveness analyses are required to model different patient pathways, and assess the costs and benefits of different STI POCTs currently in development.^11^

This service evaluation was conducted in one SHC in London, and the results are therefore not generalisable. Larger studies to evaluate the clinical impact of POCTs in clinic are required, which would in turn help to both populate the parameter inputs of cost-effectiveness models, and provide much needed clinical effectiveness data to support adoption of these technologies in SHCs.

**Key messages**

- A ‘sample first’ clinical pathway (providing samples on clinic arrival) was acceptable, enabling successful rapid and point-of-care test (POCT) deployment in a busy sexual health clinic.
- Non-nucleic acid amplification test (NAAT) POCT results were available for all patients prior to leaving clinic.
- With only one-fifth of patients receiving *Chlamydia trachomatis*/*Neisseria gonorrhoeae* NAAT results before leaving clinic, the GeneXpert was a rapid, not POC, test in our clinic.
- ‘Sample first’ deployment of multiple POCTs impacted patient management, with additional *Trichomonas vaginalis* and bacterial vaginosis diagnoses and three patients not receiving presumptive treatment.
