# Supplementary figures and images for: Impact of deploying multiple point-of-care tests with a ‘sample first’ approach on a sexual health clinical care pathway. A service evaluation
Source: Sex Transm Infect. 2017 Feb 3;93(6):424–9. doi: 10.1136/sextrans-2016-052988 (PMC5574381; doi:10.1136/sextrans-2016-052988)

# Clinical decision algorithm for making a diagnosis of Bacterial vaginosis (BV)

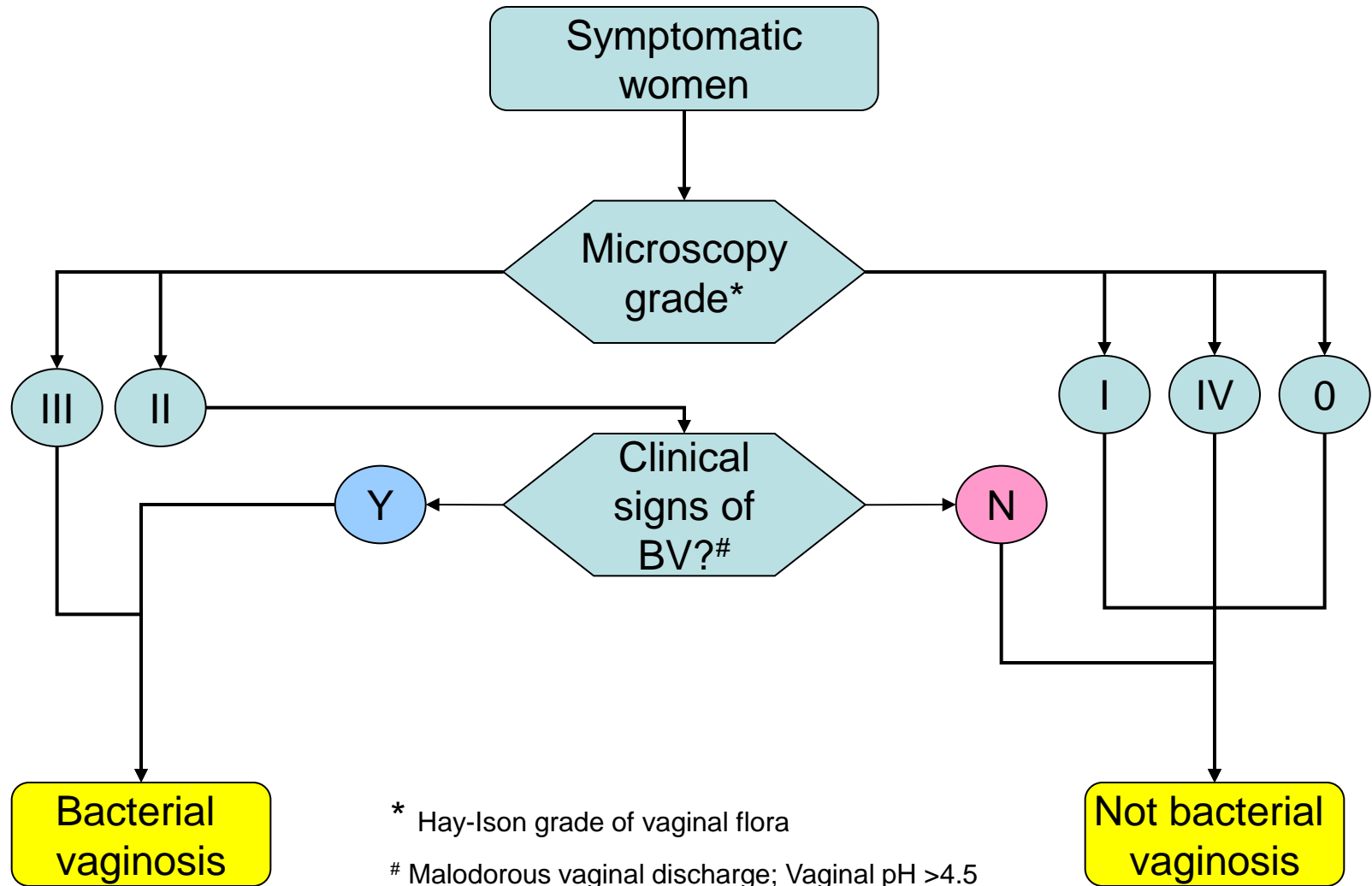

Supplement: supplementary material [file sextrans-2016-052988supp002.pdf]
